# “Diet in the Etiopathogenesis of IBD: Is There A Culinary Culprit?”

**DOI:** 10.1093/crocol/otz055

**Published:** 2020-01-16

**Authors:** Kelly Issokson

**Affiliations:** Department of Medicine, Cedars-Sinai Medical Center, Los Angeles, CA

With the recent increase in inflammatory bowel disease (IBD) incidence globally, environmental factors, such as diet, are thought to play an important role in disease onset. Ananthakrishnan presents an interesting but limited review of the evidence examining macronutrients, micronutrients, dietary components, and their influence on the development of IBD. The author proposes three mechanisms of action of diet on intestinal inflammation: 1) through direct changes in microbiota composition and diversity; 2) its effect on intestinal barrier integrity; and 3) its effect on immune response pathways.

We know from prior studies that diet influences the microbiome and metabolome. In an elegant study,^1^ De Filippo and colleagues investigated the microbiome of children living in rural Africa who ate a high-fiber diet and compared this with children living in Europe who ate a westernized diet low in fiber. They found that the microbiota of the African children was significantly richer in Bacteroidetes and poorer in Firmicutes, the former of which is necessary for the fermentation of fiber polysaccharides and the production of metabolites such as short-chain fatty acids (SCFAs) which exert an anti-inflammatory and immunoregulatory effect. Maslowski and colleagues^2^ suggest that diet-induced changes in microbiota and their metabolic by-products influence onset of inflammatory diseases, particularly in those with a genetic predisposition. This review highlights research from Ananthakrishnan et al^3^ which found fiber intake of 24 g per day to be associated with a 40% reduction in the risk of developing Crohn’s disease.

The strongest evidence the author cites that supports a link between diet and IBD onset comes from a few large prospective cohorts of mostly white- and older-aged (median age around 50 years) individuals.^3–5^ One study^4^ suggests that high ratios of omega 3 (ω3)/omega 6 (ω6) fatty acid intakes are inversely associated with IBD risk, but this appears to be strongest for decreasing risk of UC (ulcerative colitis) in females; no association was found for Crohn’s disease. Investigators analyzing data from the European Prospective Investigation into Cancer and Nutrition (EPIC) cohort^5^ found that greater concentrations of arachidonic acid (ω6) were associated with an increased risk of UC. These studies are consistent with findings from other large prospective cohorts, one of which^6^ found increased intakes of ω6 fatty acids doubled the risk for UC, but this was significant for females only and significance was lost when adjusting for intake of other fatty acids. They also found a statistically significant negative association with intake of the ω3 fatty acid docosahexaenoic acid, or DHA, and development of UC. Another large prospective cohort in the UK^7^ found DHA to be protective for developing UC.

The author also cites three observational studies that found an inverse association with predicted vitamin D levels (although they applied a model validated in males to this female cohort), zinc intake (from food, not supplements), and intakes of resveratrol (from grapes and wine) and flavones (from thyme, rosemary, oregano) and the risk of Crohn’s disease.^8–10^ Micronutrients such as vitamin D can be affected by acute and chronic inflammation, and whether low levels are a result of or a risk factor for IBD is poorly understood. We know that vitamin D regulates innate immunity, and recent laboratory studies strongly suggest that deficiencies in vitamin D signaling play a role in the development of Crohn’s disease.^11^

Finally, there is emerging evidence that the widespread use of emulsifiers (e.g., carboxymethylcellulose and polysorbate 80) contributes to metabolic syndrome and other inflammatory diseases.^12^, ^13^ Ananthakrishnan cites a rodent study that found emulsifiers altered the microbiota and increased colitis severity.^14^ Although many food additives are considered to be generally recognized as safe (GRAS) by the Food and Drug administration, their safety in human health is not well established. Future studies examining diet and IBD risk should use validated tools that are able to capture these food additives so that their effects on human health can be better understood. Until then, we know that emulsifiers do not contribute nutritional value and it is probably best to avoid them where possible.

The limitations of this review include the underrepresentation of minorities and those of a younger age in the cited cohorts. Although these large cohorts are able to analyze some diet components well (macronutrients and micronutrients), they are limited in their ability to capture dietary nuances such as food processing and food additives and thus unable to provide insight into their effects on intestinal inflammation. It is also unclear what role host genetics or baseline microbiota are playing here.

Whether diet is influencing disease risk and how is still largely unknown. Further studies are needed, including large prospective cohorts representing populations with greater diversity in gender, age, and ethnicity to help clinicians better understand the complex role of diet in IBD onset. For patients looking to reduce risk for IBD or other chronic diseases (e.g., heart disease and diabetes), current evidence suggests the following for reducing IBD risk:

- Eat less
  - ω6 polyunsaturated fatty acids from red meat, corn and sunflower oil, and margarine
  - processed foods and food additives (carboxymethylcellulose and polysorbate 80)
- Eat more
  - ω3 polyunsaturated fatty acids from fatty fish and other seafood (e.g., salmon, mackerel, tuna, herring, and sardines) and nuts and seeds (e.g., flax seed, chia, walnuts)
  - fiber; at least 24 g per day, mostly from fruits and vegetables
  - zinc-rich foods
  - Maintain adequate vitamin D levels

Referring patients to a registered dietitian (RD) can help patients develop a nutrition plan that is tailored to their individual needs. Legislation that supports healthy eating initiatives (e.g., increasing availability of fresh foods in underserved communities; expanding access to RD services to include all individuals and not just those who have already been diagnosed with chronic diseases) is essential for improving the health of our community and decreasing disease burden.

## Notes

I have no financial disclosures or conflicts of interest.
